# The Relation of the Ligamentum Nuchæ to the First Cervical Vertebra in the Horse

**Published:** 1899-05

**Authors:** G. S. Hopkins

**Affiliations:** Assistant Professor of Veterinary Anatomy and Anatomical Methods, New York State Veterinary College, Ithaca, N. Y.


### THE RELATION OF THE LIGAMENTUM NUCHÆ TO THE FIRST CERVICAL VERTEBRA IN THE HORSE.

By G. S. Hopkins, D.Sc.,
ASSISTANT PROFESSOR OF VETERINARY ANATOMY AND ANATOMICAL METHODS, NEW YORK STATE VETERINARY COLLEGE, ITHACA, N. Y..

The subject herein presented was suggested by the disparity of statement in the text-books on veterinary surgery and the absence of any definite statement in the standard veterinary anatomies with reference to the relation of the cervical ligament to the atlas, particularly the presence of a synovial bursa between the two for facilitating the gliding of the ligament upon the atlas. In the works on surgery the presence of such a bursa is most positively affirmed, but in none of the anatomies consulted (Chauveau, Franck, Leyh, Gamgee and Law, M'Fadyean, Percivall, Rigot, Strangeway) is any mention made of such a structure. It seems inexplicable that such an important structure should have been overlooked by all of these skilled anatomists, while, apparently, every surgeon found it without difficulty. In order to account for this discrepancy, if possible, observations have been made upon thirty-five subjects, of which thirty-two were adults and three were young colts only a few days old.

Before stating the results of these observations it may be well to recall briefly the relations of the occipital, or funicular, portion of the ligamentum nuchæ to the surrounding structures.

Superficially this portion of the ligament is covered by the skin, the cervico-auricularis muscles, and a mass of fibro-fatty tissue. On each side is the muscle rectus capitis posticus major, and the tendons of the complexus major and splenius, while between the ligament and the atlas is the muscle rectus capitis posticus minor. The ligament is rather loosely joined to the muscles by fine areolar connective tissue; but in the median plane there is, in some specimens, a narrow but well-marked fibrous septum passing from between the two lateral portions of the ligament to the underlying muscles and the capsular ligament of the occipito-atloid articulation.

Let us now for a moment note what is said concerning this bursa in some of the works on surgery. Hoffman (*Surgery*, vol. ii. p. 262) says that between the first and second cervical vertebræ and the ligament there is a mucous bursa. In Möller's *Surgery* (translated by Dollar) is this statement: "In the horse the funicular portion of the ligamentum nuchæ forms on the summit of the second cervical vertebra a mucous bursa which attains the size of an apple and is covered on both sides of the ligament by the complex muscles. Loose connective tissue attaches the inner surfaces of these muscles to the bursa." Lafosse (*Recueil de Med. Vet.*, tome lii. p. 329), in describing the anatomical relations of the ligament in this region, says: "Its gliding on the atlas and the atloido-axoid articulation is facilitated by a mucous bursa . . . and is replaced when it fails by a laminated connective tissue with large lacunæ." Peuch (*Journal de Med. Vet.*, tome xlvii. p. 70) says that this cavity "is developed in the fibro-elastic tissue interposed between the cervical ligament on the one hand and the atloido-occipital ligament on the other. It is formed at the expense of this tissue, and also from the serous bursa, which facilitates the gliding of the cord of the cervical ligament on the arch of the atlas and which, scarcely noticeable in the young subject, increases in size with age."

In the thirty-five cases which I have examined conditions were found which would seem to account for the descriptions of this region as given both in the text-books of anatomy and of surgery. Of the thirty-two adult horses examined no bursa could be found in ten of them, while in twenty-two a distinct cavity was present between the occipital portion of the ligament and the atlas. In the three young colts no bursa could be distinguished. Of the ten adults in which no bursa was found, the oldest one of which I have any record was thirteen years of age. Another was eight years old; one was between four and five years, and one about one and onehalf years old. In all these ten, together with the three colts, that portion of the ligament passing over the atlas was perfectly smooth and showed no signs whatever of abrasion, nor could any differences be detected in the appearance of the connective tissue of this region from that farther down the neck.

In a large majority of the twenty-two subjects in which a bursa was found its presence appeared to be due to pathological changes rather than to normal conditions. In eighteen instances there were either calcareous deposits in the walls of the sac or the ligament was more or less roughened and abraded, in some instances being cut nearly half in two, although more often the two conditions coexisted. In four instances only were no indications found of calcareous deposits or abrasions of the cervical ligament. One of these four had many trabeculæ extending through it, but the other three were simple sacs, with no suggestion of pathological origin, as in the other cases. The size of this subnuchal space varied in length from five to fourteen centimetres; its average length was about eight centimetres. In most cases the space was exceedingly irregular and roughened, either by fibrous outgrowths or nodules of calcareous material, which, for the most part, were firmly adherent to the surrounding tissues.

Judged simply by the above facts, it would appear that normally there is no serous bursa between the ligamentum nuchæ and the atlas, but that the large sac often present in this region is due, apparently, to secondary changes of a pathological nature.

Summary:

| | Adults. | Colts. |
|---|---|---|
| No bursa present, ligament perfectly smooth . . . | 10 | 3 |
| Bursa present . . . . . . . . . . | 22 | |
| Bursa with calcareous deposits or abraded ligament . | 18 | |
| Bursa present and ligament perfectly smooth . . | 4 | |

